# Integrative analysis of single-cell and bulk RNA-sequencing data revealed disulfidptosis genes-based molecular subtypes and a prognostic signature in lung adenocarcinoma

**DOI:** 10.18632/aging.205509

**Published:** 2024-02-05

**Authors:** Haixia Wang, Xuemei Zhu, Fangchao Zhao, Pengfei Guo, Jing Li, Jingfang Du, Guoyong Shan, Yishuai Li, Juan Li

**Affiliations:** 1 Department of Radiation Oncology, The Fifth Clinical Medical College of Henan University of Chinese Medicine, Zhengzhou People’s Hospital, Zhengzhou 450003, China; 2 Department of Ultrasound, Jurong Hospital Affiliated to Jiangsu University, Zhenjiang 212000, China; 3 Department of Thoracic Surgery, The Second Hospital of Hebei Medical University, Shijiazhuang 050000, China; 4 Department of Clinical Medicine, Hebei University of Engineering, Handan 056002, China; 5 Department of Thoracic Surgery, Hebei Chest Hospital, Shijiazhuang 050000, China; 6 School of Nursing, Shandong First Medical University and Shandong Academy of Medical Sciences, Taian 271000, China

**Keywords:** disulfidptosis, lung adenocarcinoma, tumor microenvironment, immune checkpoint inhibitors, chemoradiotherapy

## Abstract

Background: Disulfidoptosis is an unconventional form of programmed cell death that distinguishes itself from well-established cell death pathways like ferroptosis, pyroptosis, and necroptosis.

Methods: Initially, we conducted a single-cell analysis of the GSE131907 dataset from the GEO database to identify disulfidoptosis-related genes (DRGs). We utilized differentially expressed DRGs to classify TCGA samples with an unsupervised clustering algorithm. Prognostic models were built using Cox regression and LASSO regression.

Results: Two DRG-related clusters (C1 and C2) were identified based on the DEGs from single-cell sequencing data analysis. In comparison to C1, C2 exhibited significantly worse overall prognosis, along with lower expression levels of immune checkpoint genes (ICGs) and chemoradiotherapy sensitivity-related genes (CRSGs). Furthermore, C2 displayed a notable enrichment in metabolic pathways and cell cycle-associated mechanisms. C2 was also linked to the development and spread of tumors. We created a prognostic risk model known as the DRG score, which relies on the expression levels of five DRGs. Patients were categorized into high-risk and low-risk groups depending on their DRG score, with the former group being linked to a poorer prognosis and higher TMB score. Moreover, the DRG score displayed significant correlations with CRSGs, ICGs, the tumor immune dysfunction and exclusion (TIDE) score, and chemotherapeutic sensitivity. Subsequently, we identified a significant correlation between the DRG score and monocyte macrophages. Additionally, crucial DRGs were additionally validated using qRT-PCR.

Conclusions: Our new DRG score can predict the immune landscape and prognosis of LUAD, serving as a reference for immunotherapy and chemotherapy.

## INTRODUCTION

Lung cancer is a widespread malignant disease globally, being the primary cause of cancer-related fatalities. This is mainly attributed to its aggressive growth and tendency to spread to distant sites [1]. At present, lung adenocarcinoma (LUAD) is the most common histological subtype among lung malignancies. Despite recent advances in comprehensive treatments, the challenge of metastasis still significantly affects the achievement of favorable clinical outcomes [2]. Numerous therapeutic modalities, particularly immunotherapy, have recently emerged as essential components of treatment protocols, demonstrating strong protective efficacy for individuals with LUAD [3]. Despite patients having comparable clinicopathological characteristics, it is evident that there are significant variations in drug responses. This indicates that traditional classifications, such as the TNM staging system, are insufficient for predicting therapeutic outcomes [4, 5]. To address this limitation, it is crucial to identify innovative molecular characteristics capable of efficiently categorizing LUAD patients into distinct subgroups with a greater likelihood of responding to targeted therapeutic interventions.

The etiology and progression of LUAD are closely tied to the abnormal regulation of various cellular death pathways, including apoptosis, necrosis, autophagy, and ferroptosis, among others [6]. Recently, a research team has discovered and identified a new form of cell death called disulfidptosis, which provides new opportunities for cancer treatment. Disulfidptosis is a type of cellular death that occurs due to an excessive accumulation of disulfide linkages within the cellular environment. This phenomenon leads to the destabilization and eventual breakdown of the actin filament network. Disulfidptosis primarily relies on the expression of the SLC7A11 protein, which is involved in both glutathione synthesis and transport, thus influencing the cell’s redox balance. Moreover, this study highlights that using inhibitors of glucose transporters induces disulfidptosis in cancer cells with high SLC7A11 expression, effectively suppressing the growth of SLC7A11-overexpressing tumors [7]. Recognizing disulfidptosis as a distinctive cell death mechanism offers a vital foundation for understanding and addressing this process in cancer therapy. Given the complex interplay, disulfidptosis emerges as a promising and distinctive molecular hallmark, requiring thorough investigation in future research efforts. Nonetheless, it remains uncertain whether disulfidptosis plays a pivotal role in LUAD, and additional research is needed to investigate this aspect.

We conducted a thorough bioinformatics analysis of disulfidptosis-related genes (DRGs) in LUAD, utilizing publicly accessible datasets. We evaluated their expression patterns, tumor microenvironment (TME) infiltration, prognostic significance, and potential molecular mechanisms in LUAD. Our findings provide new insights into understanding the molecular basis of disulfidptosis in LUAD and have significant implications for its diagnosis and therapeutic strategies.

## MATERIALS AND METHODS

### Single cell sequencing data download and processing

We downloaded the LUAD single-cell dataset GSE131907 from the GEO database, which consists of 11 samples. Our subsequent procedure involved performing quality control of the data. Specifically, we retained cells that showed a low proportion of mitochondrial genes (less than 10%), as well as those with an excessive total number of genes (more than 200), and genes exhibiting expression levels between 200 and 7000, and were expressed in fewer than three cells. Eleven samples underwent SCT correction for integration. Subsequently, we utilized the tSNE technique to reduce data dimension by setting the “DIMS” parameter to 20. Additionally, we set the resolution to 1.0 using the KNN clustering algorithm for cell clustering. Afterward, a range of cell surface markers was used for cellular annotations. Ultimately, by importing DRGs using the “PercentageFeatureSet” function, we can determine the percentage of DRGs in each cell.

### Bulk RNA-seq data collection

The bulk tumor transcriptomic data from the TCGA and the clinical records of patients with LUAD were obtained from the UCSC Xena platform (https://xenabrowser.net/). These datasets were used to identify genes associated with patient survival and to create prognostic signatures. Four distinct microarray datasets, namely GSE30219, GSE50081, GSE26939, and GSE72094, were obtained from the GEO database for external validation in this study.

### Unsupervised clustering for DRG

In previous research, we carefully extracted a total of ten DRGs and presented the gene details in Supplementary Table 1. In our study, we conducted a thorough unsupervised clustering analysis based on consensus, using the “ConsensusClusterPlus” R package. This analysis was conducted using a carefully defined set of parameters, including maxK = maxK, reps = 50, pItem = 0.8, pFeature = 1, clusterAlg = “km”, distance = “Euclidean”, and seed = 123456. The main goal of this analysis was to efficiently categorize patients into specific DRG clusters, relying on the evaluation of their DRG expression profiles. Furthermore, we conducted a principal component analysis (PCA) to effectively illustrate the categorization of the DRG clusters. Subsequently, we compared the overall survival (OS) probabilities of the DRG clusters using the R package “survival”.

### Correlations of DRG clusters with chemoradiotherapy sensitivity–related genes (CRSGs), immune checkpoint genes (ICGs) and tumor microenvironment (TME)

After retrieving CRSGs and ICGs, we analyzed their differential expression in DRG clusters. We used the R package “ESTIMATE” to calculate TME scores, which include stromal, immune, and estimated scores based on gene expression profiles.

### Gene set variation analysis (GSVA) and gene set enrichment analysis (GSEA)

To investigate potential differences in biological functions among DRG clusters, we used the R package “GSVA” to conduct GSVA analysis with the “c2.cp.kegg.v7.5.symbols” and “c5.go.bp.v7.5.symbols” gene sets. To visualize the results, we used the R package “pheatmap”. We conducted GSEA using the “clusterProfiler” R package in combination with the hallmark gene set “h.all.v7.2.symbols” obtained from MSigDB.

### Identification of differentially expressed genes (DEGs) between DRG clusters and functional annotation

We used the R package “limma” to identify the DEGs within the distinct DRG clusters. The selection criteria for the identification of DEGs were defined as having an absolute |log2 (FoldChange)| exceeding 0.5, coupled with a stringent adjusted *p*-value threshold of less than 0.05. Additionally, to uncover the underlying biological mechanisms linked to these DEGs in DRG clusters, we conducted enrichment analyses using the Gene Ontology (GO) and Kyoto Encyclopedia of Genes and Genomes (KEGG) databases, and utilized the “clusterProfiler” package.

### Construction and evaluation of the DRG prognostic model

In the TCGA-LUAD cohort, patients were randomly allocated, ensuring an even distribution between the training and internal testing cohorts with a 1:1 ratio, facilitated using the “caret” R package. To reduce the complexity of the high-dimensional data associated with DEGs linked to OS, we utilized the Least Absolute Shrinkage and Selection Operator (LASSO) Cox regression technique, implemented using the “glmnet” R package. Specifically, we utilized the “cv.glmnet” function with predefined parameters, specifying the type.measure as “deviance” and the alignment as “lambda”. We conducted ten-fold cross-validation to reduce overfitting risk, while selecting the optimal penalty parameter (λ) based on a strict criterion of minimal value. We performed a multivariate Cox regression analysis to identify potential genes from the pool of candidate genes. We used the “My.stepwise.coxph” function with parameters, including sle = 0.15, sls = 0.15, and vif.threshold = 999. We then proceeded to construct the prognostic model for DRG within the training cohort. We formulated a prognostic scoring framework for individuals with LUAD, using the median value of the predicted DRG score as the threshold for classification. Subsequently, patient cohorts were categorized into two distinct risk groups: high-risk (DRG score > median) and low-risk (DRG score < median), based on this specific threshold. We utilized the R packages “survival” and “survminer” to conduct Kaplan-Meier analysis and compare survival probabilities between the two groups. Additionally, we employed the “timeROC” R package to conduct receiver operating characteristic (ROC) analysis for prognostic predictions over 1, 3, and 5 years. Subsequently, we calculated the area under the curve (AUC) for these analyses.

### Independent prognostic analysis and establishment of a nomogram

We collected clinical data for LUAD patients from the TCGA dataset. To personalize the predicted survival probability for LUAD patients, we developed a nomogram incorporating clinical characteristics and DRG score. We assessed the accuracy of the nomogram’s predictions through calibration plot analysis, and used decision curve analysis (DCA) to determine their clinical usefulness.

### Correlations of DRG score with tumor mutational burden (TMB), ICGs, and immunotherapy response

Patient response to immunotherapy has been associated with both TMB and ICGs. To gain insights into the mutational landscape of LUAD patients across various DRG score groups, we employed the “maftools” R package to extract the Mutation Annotation Format (MAF) from the TCGA database. Additionally, TMB scores were calculated for the TCGA-LUAD cohort. Subsequently, we used Spearman’s rank correlation coefficient to assess the relationships between ICGs and the DRG score, as well as the five genes that make up the DRG prognostic model. To assess the effectiveness of immunotherapy for LUAD patients, we utilized the tumor immune dysfunction and exclusion (TIDE) algorithm, which can be found at http://tide.dfci.harvard.edu/. The algorithm offers valuable insights that can aid doctors in identifying patients who are more likely to respond positively to immunotherapy [8].

### Correlations of DRG score with CRSGs and chemotherapeutic sensitivity

We obtained relevant CRSGs from previous research (Supplementary Table 2). Subsequently, we assessed the expression profiles of CRSGs in different risk categories and investigated the correlation between DRG scores and gene expression levels. To calculate drug sensitivity scores related to the standard treatment for LUAD, we used the “calcPhenotype” function provided by the “oncoPredict” R package. Lower drug sensitivity scores indicate increased responsiveness to the medication.

### Protein expression validation

Immunohistochemistry (IHC) Validation: We used immunohistochemical staining images from the Human Protein Atlas (HPA) database, available at v19.3.proteinatlas.org [9], to confirm the expression of the critical genes under investigation in both LUAD and adjacent normal tissue specimens. The evaluation of IHC images in the HPA database involved a comprehensive assessment of staining characteristics, intensity, quantity, and location with regard to individual genes (detailed information about the statistical methods used for analyzing IHC images is available at the following web address: https://www.proteinatlas.org/about/assays+annotation#ih_annotation).

### Quantitative real-time polymerase chain reaction

The cell lines utilized in this study, namely LUAD cell lines (A549, H1299, and HCC827), in addition to the human normal bronchial epithelial cell line (BEAS2B), were generously sourced from the Cell Repository of the Chinese Academy of Sciences, situated in Shanghai, China. All cell lines were diligently maintained in RPMI-1640 medium, supplemented with 10% Fetal Bovine Serum (FBS), streptomycin (100 U/mL), and penicillin (100 U/mL), and incubated at a temperature of 37°C under a controlled atmosphere of 5% CO_2_.

TRIzol^®^ (1 mL) was employed to extract total RNA from the cell lines. Subsequently, complementary DNA (cDNA) synthesis was accomplished utilizing reverse transcriptase derived from avian medulloblastoma virus, in accordance with the guidelines provided by TAKARA. For the quantification of gene expression via qRT-PCR, SYBR Premix Ex Taq II, a product of Takara located in Shiga, Japan, was utilized. Data analysis was carried out utilizing the 2^−ΔΔCT^ method. Detailed information regarding the primer sequences utilized for the qRT-PCR analysis is presented in Supplementary Table 3.

### Statistical analysis

The statistical analyses in this study were performed utilizing R software, specifically version 4.0.1, as previously detailed. Significance was established at a threshold of *p* < 0.05, denoting statistical significance.

## RESULTS

### Single cell sequencing data analysis

**Figure 1:**
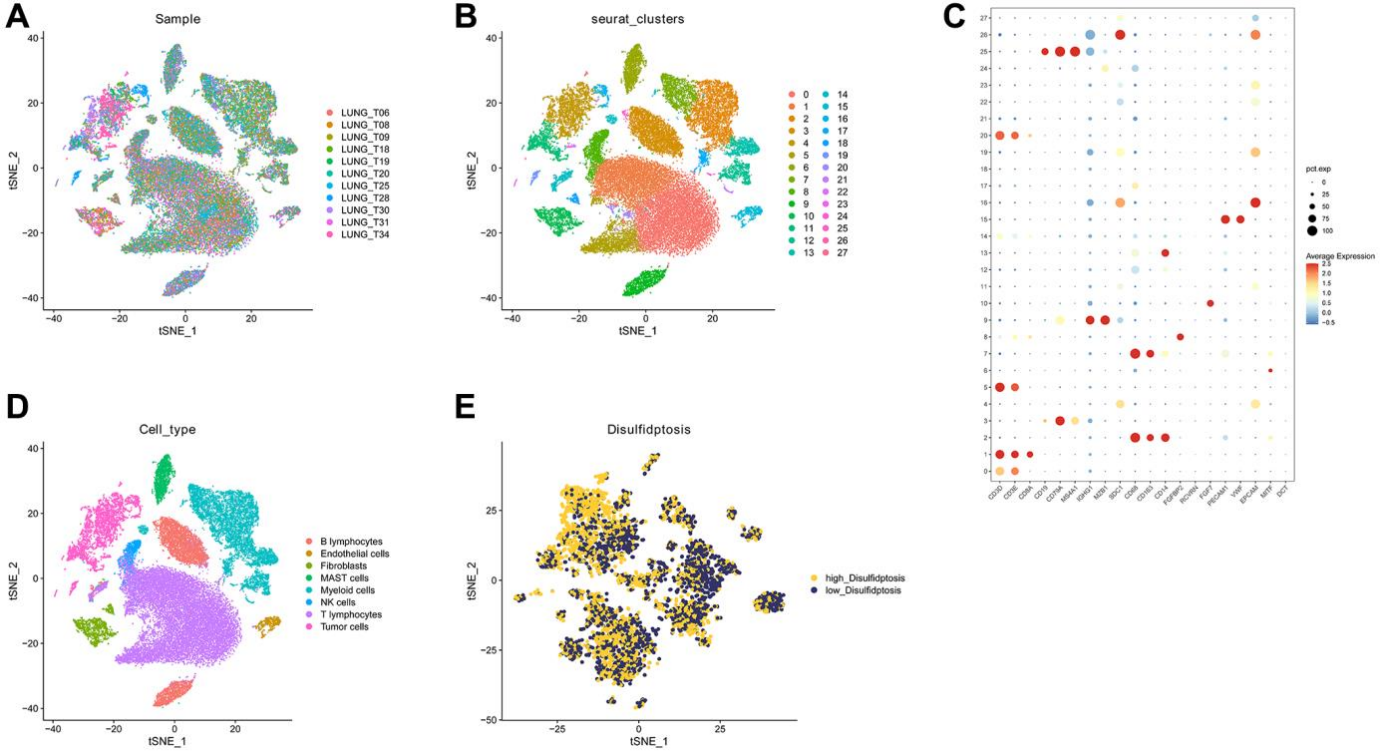
**Analysis of GSE131907 using single-cell sequencing technology.** (**A**) The 11 samples exhibit a notable level of integration efficacy. (**B**, **C**) Dimensionality reduction and cluster analysis. (**D**) According to the surface marker genes of different cell types, the cells are annotated as BB lymphocytes, endothelial cells, fibroblasts, MAST cells, myeloid cells, NK cells, T lymphocytes, and tumor cells, respectively. (**E**) The cells were divided into high- and low-disulfidptosis cells according to the percentage of DRG in each cell.

Initially, we conducted an in-depth analysis of the single-cell sequencing dataset GSE131907 specific to LUAD, with the objective of integrate disparate samples within this dataset. Our analysis, illustrated in Figure 1A, revealed that the integration of 11 samples was effective, and no apparent batch effect was present, indicating its suitability for further analysis. We employed the KNN clustering algorithm to group all the cells into 28 distinct clusters (Figure 1B). Later, we used the surface marker genes and their expression levels for various cell types to ultimately identify eight distinct cell types (Figure 1C). These cell types include B lymphocytes, endothelial cells, fibroblasts, NK cells, myeloid cells, T lymphocytes, MAST cells, and tumor cells, as shown in Figure 1D. Subsequently, we extracted tumor cells and utilized the “PercentageFeatureSet” function to input 10 DRGs, which enabled us to determine the percentage of DRGs present in each cell. The cellular populations were categorized into two distinct groups, namely cells exhibiting low and high levels of disulfidptosis, as determined by their respective median DRG proportions. Subsequently, these data were visualized through the generation of tSNE diagrams (Figure 1E). Further analysis identified 1,537 DEGs between the high-disulfidptosis and low-disulfidptosis groups.

### Correlations of DRG clusters with CRSGs, ICGs and TME

**Figure 2:**
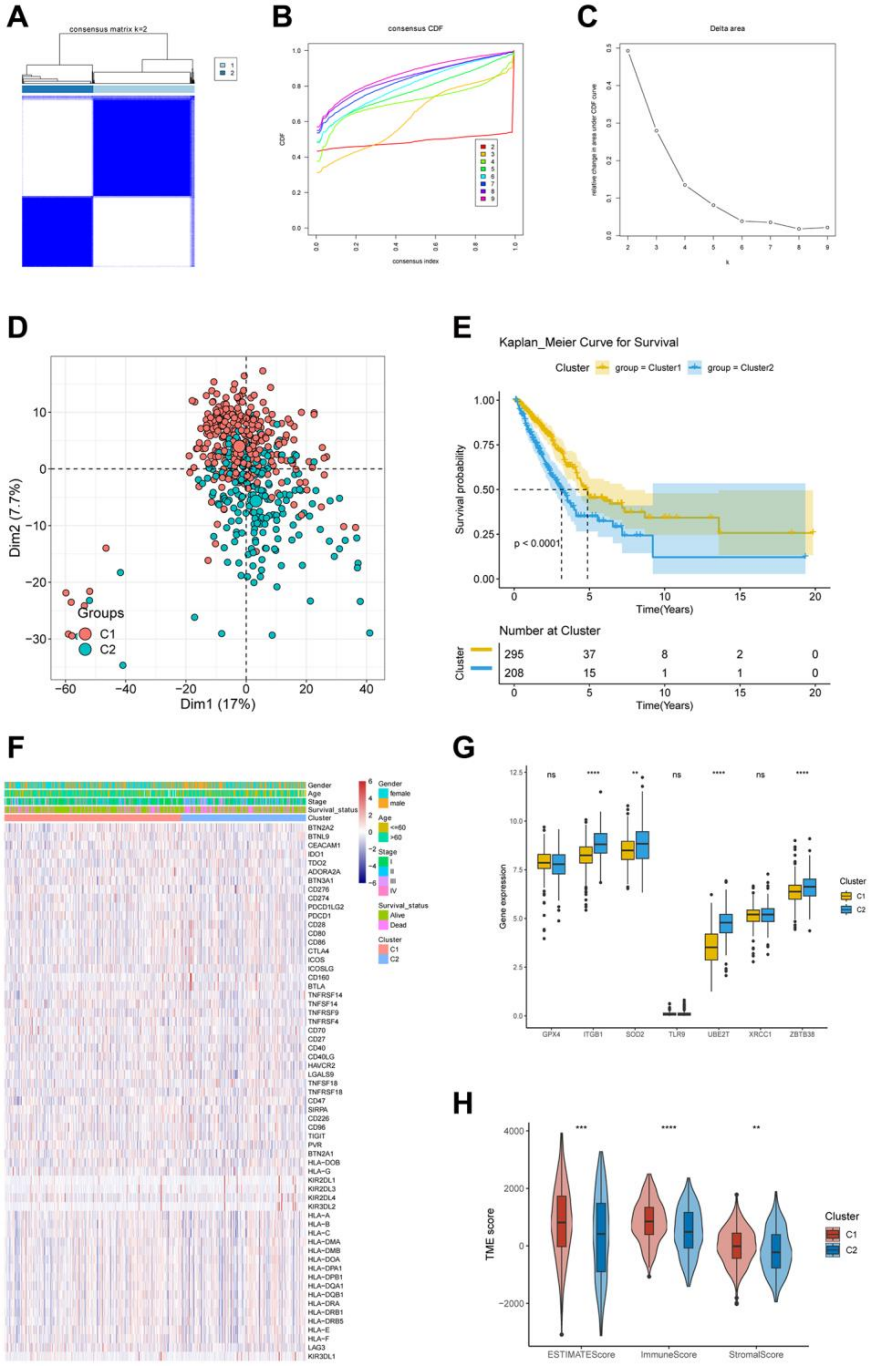
**Correlations of DRG clusters with CRSGs, ICGs, and TME.** (**A**) The TCGA-LUAD cohort was partitioned into two distinct clusters based on the consensus clustering matrix (k = 2). (**B**) Uniform clustering CDF with k from 2 to 9. (**C**) The alteration in the area beneath the CDF curve as the value of k varies from 2 to 9. (**D**) The PCA plot showed the distribution of samples among 2 DRG clusters. (**E**) Kaplan-Meier survival analysis for two DRG clusters. (**F**) The heatmap analysis revealed distinct patterns of ICGs expression, as well as clinicopathological features, that distinguished DRG cluster 1 from DRG cluster 2. (**G**) The differential expression of seven CRSGs between two distinct clusters of DRGs. (**H**) Exploring the correlations between CRG clusters and TME scores. ^*^P < 0.05, ^**^P < 0.01, ^***^P < 0.001, ^****^P < 0.0001.

To investigate the expression profiles and potential biological characteristics of DRG in the context of LUAD, we conducted a consensus clustering analysis on the TCGA-LUAD cohort. We employed 1,537 DEGs derived from the analysis of single-cell sequencing data to categorize patients into two clusters, labeled as C1 (*n* = 295) and C2 (*n* = 208) (Figure 2A–2C). A distinct variation in distribution among DRG clusters was observed in the PCA plot (Figure 2D). Moreover, we performed a Kaplan-Meier analysis to compare clinical outcomes between the two DRG clusters (Figure 2E). The results demonstrated that the overall survival rate for C2 patients was significantly lower than for C1 patients (*p* < 0.0001). Subsequently, we investigated the interrelation between DRG clusters and ICGs, CRSGs, and the TME. Our analysis indicated that C1 had a higher expression of ICGs (Figure 2F). Additionally, we detected various differentially expressed CRSGs between the two DRG clusters, such as ITGB1, SOD2, UBE2T, and ZBTB38 (Figure 2G). To explore the potential role of DRGs in LUAD immune infiltration, we employed the “ESTIMATE” and “ssGSEA” algorithms to compare TME scores between the two DRG clusters. Importantly, we noticed that patients in C1 had markedly higher TME scores than those in C2 (Figure 2H).

### Identification of differentially expressed genes between DRG clusters and functional annotation

**Figure 3:**
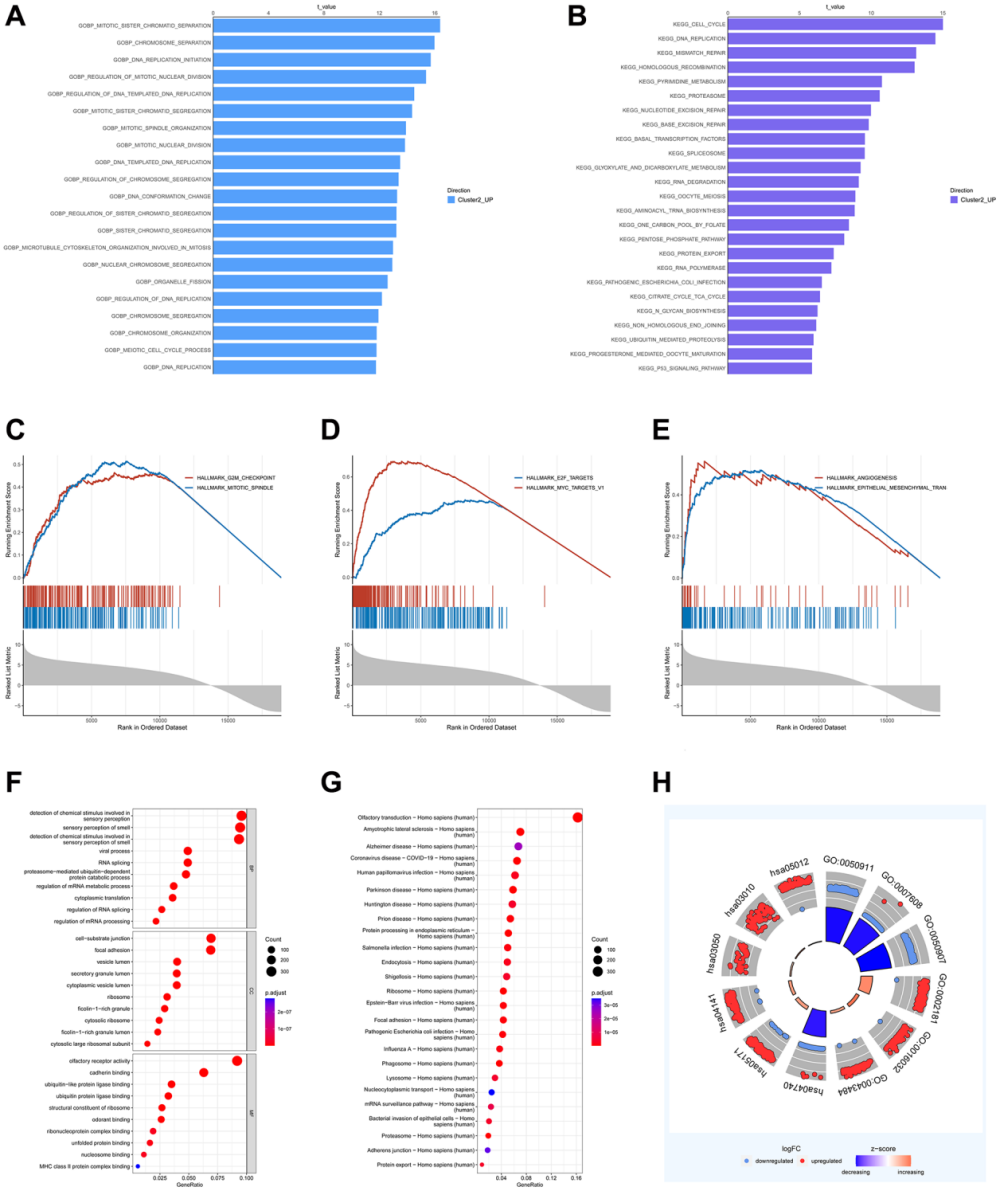
**Identification of DEGs between DRG clusters and functional annotation.** (**A**, **B**) GSVA analysis of DRG cluster 2. (**C**–**E**) GSEA of significant HALLMARK enriched in DRG cluster 2. (**F**–**H**) GO and KEGG enrichment analyses of DEGs between two DRG clusters.

To further explore the functional annotation of C1 and C2, we conducted GSVA and GSEA analyses. The results of GSVA showed that C2 had significant enrichment in cell cycle-associated processes, including DNA replication initiation, regulation of mitotic nuclear division, and organelle fission (Figure 3A). Furthermore, C2 exhibited a high abundance of pathways related to metabolism (e.g., glyoxylate and dicarboxylate metabolism, citrate cycle, TCA cycle), cell cycle regulation, and genomic stability, as shown in Figure 3B. The results from GSEA indicated that C2 is primarily associated with tumorigenesis and metastasis. This association includes hallmark features like epithelial-mesenchymal transition and angiogenesis, as depicted in Figure 3C–3E. In summary, we believe that C2’s poor survival and immune status are linked to the regulation of pathways associated with cancer.

Using the R package “limma”, we identified a total of 1,421 DEGs associated with the DRG cluster. Consistent with the results of GSVA and GSEA, the outcomes of the GO and KEGG investigations have revealed that the DEGs under examination are primarily linked to important processes, including the cell cycle, genomic stability, and cancer. This emphasizes the significant role of disulfidptosis in the complex mechanisms of tumorigenesis and metastatic progression (Figure 3F–3H).

### Construction and evaluation of the DRG prognostic model

**Figure 4:**
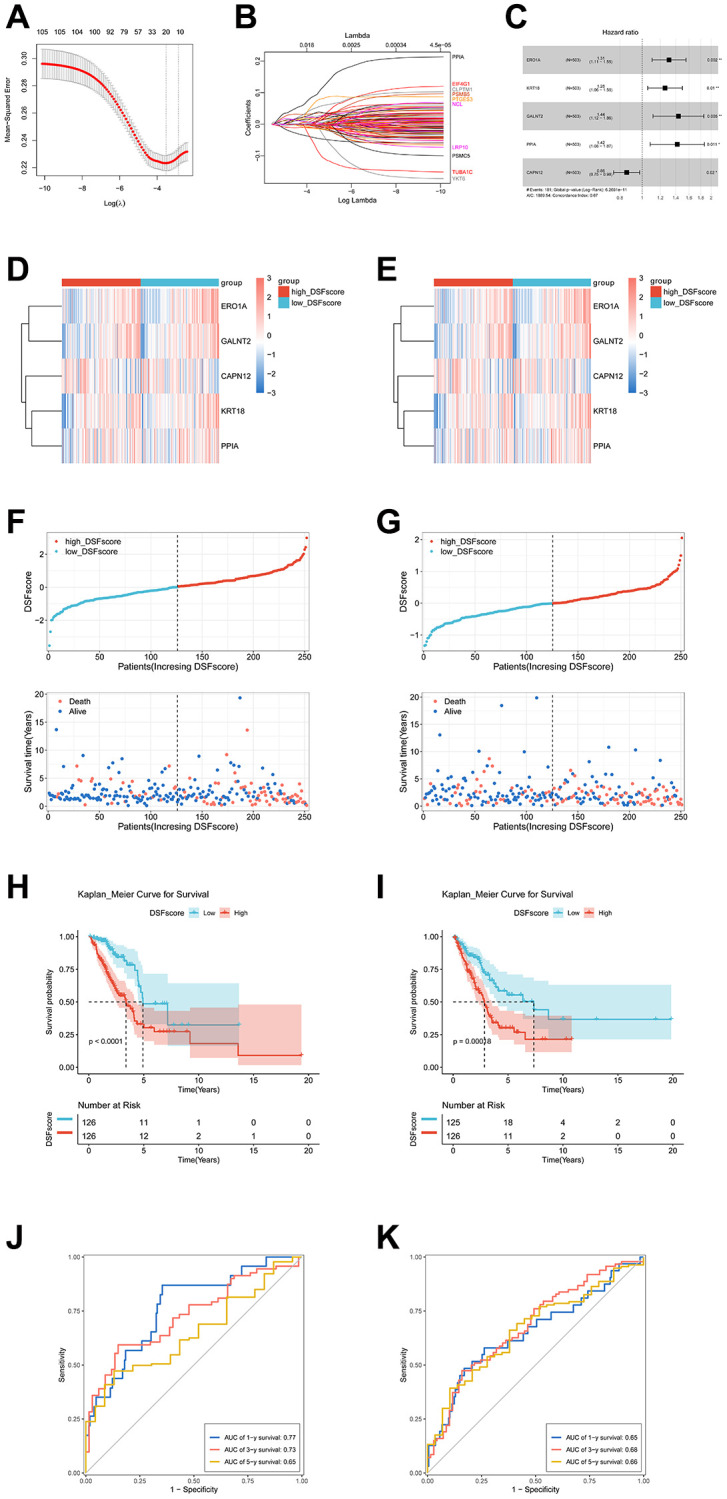
**Development and assessment of a DRG-based prognostic model in the TCGA population.** (**A**) Cross-validation for selecting the tuning parameter (λ) in the LASSO model. (**B**) The coefficient profile of prognostic DRGs using the LASSO method. (**C**) The multivariate Cox regression analysis of DRGs was presented through a forest plot. (**D**, **E**) The heatmap illustrates the differential gene expressions within the DRG prognostic model between the high- and low-risk cohorts in both the TCGA training and testing cohorts. (**F**, **G**) Risk score distribution plot and risk point plot between the high- and low-risk groups in TCGA training and testing cohorts. (**H**, **I**) The Kaplan-Meier OS curves for patients in two risk groups in TCGA training and testing cohorts. (**J**, **K**) ROC curves showed the prognostic performance of the DRG prognostic model in TCGA training and testing cohorts.

We used LASSO and multivariate Cox regression analyses on 1,421 DEGs associated with DRG clusters to create a DRG prognostic model. The TCGA cohort was split into training and testing cohorts, at a 1:1 ratio. After performing LASSO Cox regression analysis, the TCGA training cohort identified 11 genes, which were then analyzed using multivariate Cox regression. In the end, we discovered five significant genes, namely ERO1A, KRT18, GALNT2, PPIA, and CAPN12 (Figure 4A–4C). The heatmap displayed unique expression profiles of ERO1A, KRT18, GALNT2, PPIA, and CAPN12 in the high- and low-risk groups of the TCGA training and testing cohorts (Figure 4D, 4E). Additionally, we noticed that patients with higher DRG scores experienced increased mortality rates and shorter overall survival, as shown in the DRG risk plot (Figure 4F, 4G). The Kaplan-Meier analysis showed a significant difference in overall survival between high-risk and low-risk group patients (Figure 4H, 4I). In the TCGA training cohort, the AUC exhibited predictive capability for one-year, three-year, and five-year overall survival, with values of 0.77, 0.73, and 0.65, respectively (Figure 4J). In the TCGA validation cohort, the AUC values indicating predictive performance for OS at 1, 3, and 5 years were 0.65, 0.68, and 0.66, respectively (Figure 4K).

To validate the predictive accuracy of the model, we conducted an assessment using four independent external validation cohorts: GSE30219, GSE50081, GSE72094, and GSE26939. Afterward, patients in these cohorts were categorized into high- and low-risk groups based on their DRG scores. The Kaplan-Meier analysis showed a significantly better prognosis in the low-risk cohort compared to the high-risk cohort (Supplementary Figure 1A–1D). At the same time, the model demonstrated a notably high AUC value when evaluated in external validation cohorts (Supplementary Figure 1E–1H).

### Establishment and evaluation of a corresponding nomogram

**Figure 5:**
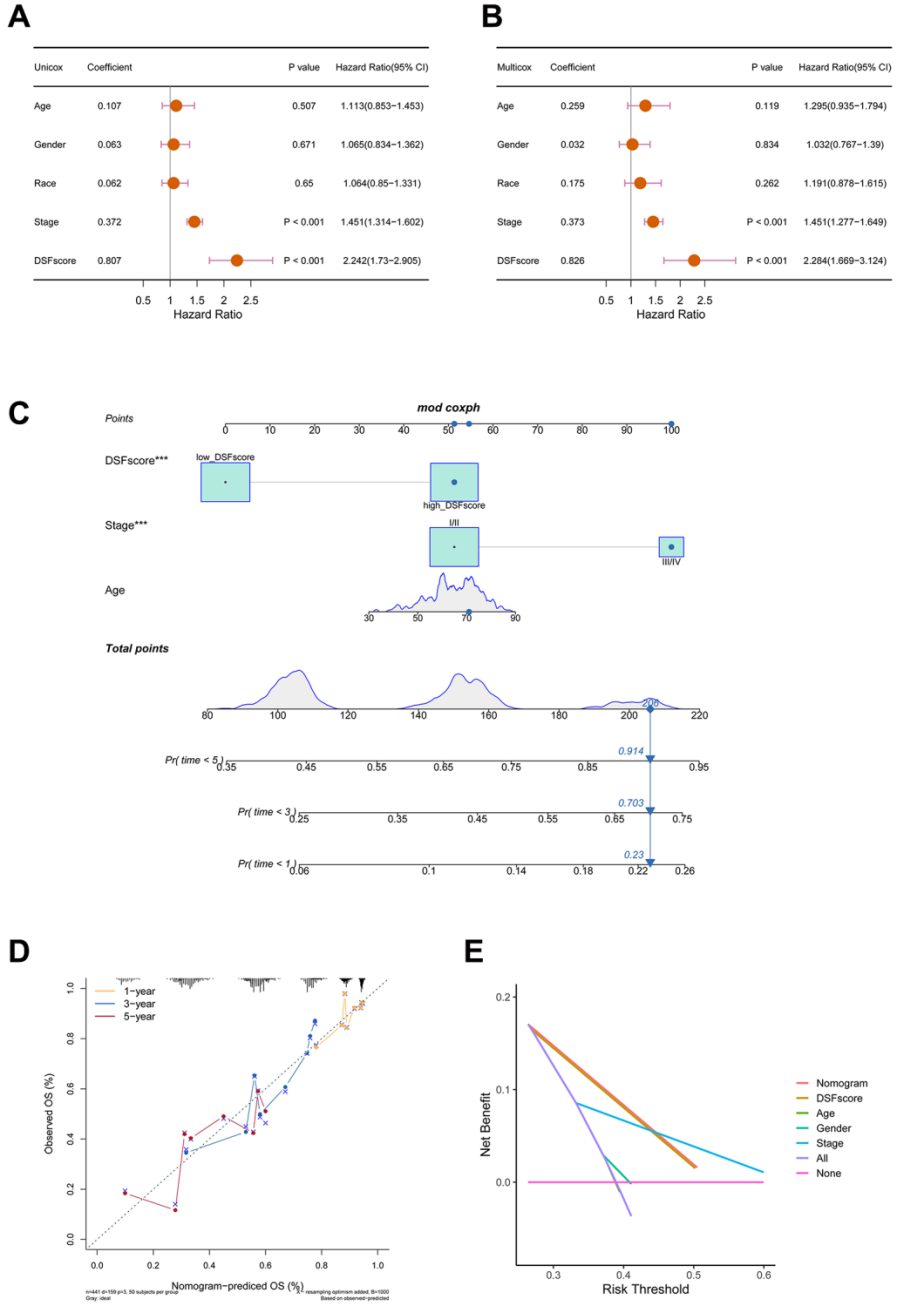
**Construction and validation of a nomogram.** (**A**) Univariate Cox regression analysis of the DRG score and clinical characteristics in the TCGA cohort. (**B**) Multivariate Cox regression analysis of the DRG score and clinical characteristics in the TCGA cohort. (**C**) Using DRG score and other clinical features to construct prognostic nomogram in the TCGA cohort. (**D**) Calibration curves of the nomogram. (**E**) DCA indicated that the nomogram achieved the optimal clinical benefit. ^***^P < 0.001.

To evaluate the DRG score’s potential as a sole prognostic indicator for OS, we integrated clinical parameters with the DRG score and then conducted univariate and multivariate Cox regression analyses. In the TCGA cohort, both stage and DRG score can be used as independent predictors of OS in LUAD patients (Figure 5A, 5B). We subsequently developed a nomogram in the TCGA cohort that incorporates DRG score, age, and stage. This nomogram provides a quantitative approach for personalized predictions of LUAD patients (Figure 5C). The calibration curves for 1, 3, and 5 years were elegantly depicted in Figure 5D. By employing DCA, we observed that the nomogram surpassed individual independent prognostic factors in providing clinical benefits for predicting the prognosis of LUAD patients (Figure 5E). In summary, our findings strongly substantiate the clinical relevance of the nomogram for predicting the prognosis of LUAD patients.

### Correlations of DRG score with TMB and ICGs

**Figure 6:**
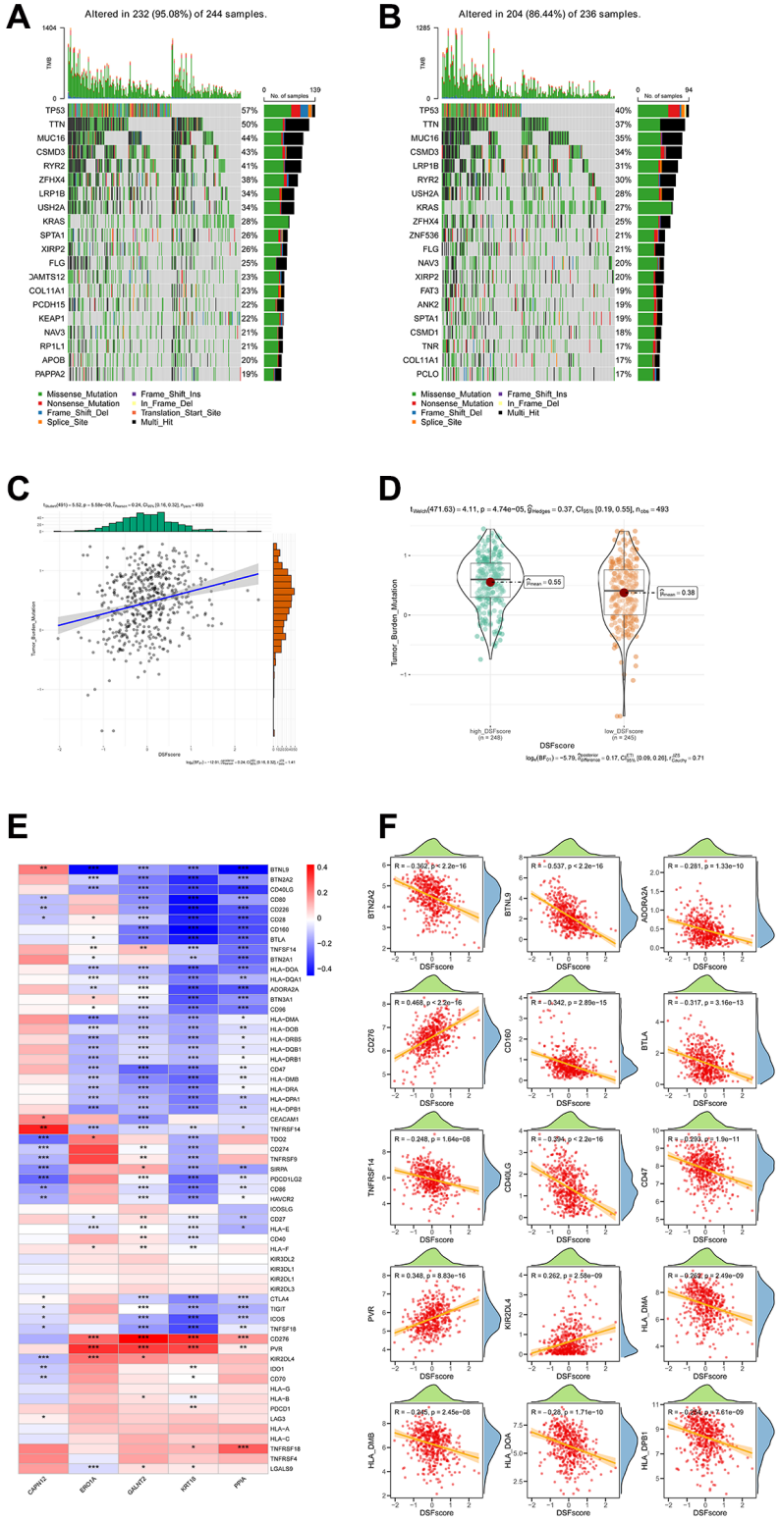
**Associations between the DRG score and TMB as well as ICGs.** (**A**, **B**) A comparative analysis of mutational profiles in two risk groups of LUAD. (**C**) Correlations between TMB and the DRG score within various gene clusters. (**D**) Disparities in TMB score among high-risk and low-risk groups. (**E**) Correlations between the expression of ICGs and the five genes included in the DRG prognostic model. (**F**) Correlations between the expression of ICGs and the DRG score. ^*^P < 0.05, ^**^P < 0.01, ^***^P < 0.001.

The mutational landscape of various DRG subgroups was depicted with waterfall plots (Figure 6A, 6B). In the entire dataset, TP53, CTNNB1, and TTN were the most commonly mutated genes in LUAD. Missense mutations were the most common type of somatic mutations. The high-risk group showed a higher prevalence of genetic mutations. Furthermore, patients with high DRG scores were discovered to be more likely to carry significant gene mutations, especially in TP53. Previous studies have clearly shown a link between higher TMB scores and increased responsiveness to immunotherapeutic treatments. In our analysis, we found a notably higher TMB score in the high-risk group (*p* = 4.74e-0.5), and we also identified a positive correlation between DRG score and TMB score (*p* = 5.58e-08) (Figure 6C, 6D). Based on previous evidence showing the relationship between ICG expression profiles and clinical responses to checkpoint blockade immunotherapy, our study conducted a thorough analysis of the correlation between the DRG score and ICGs. Our analysis uncovered significant correlations between the five genes in the model and the majority of ICGs (Figure 6E, 6F).

### Estimation of the DRG prognostic model in immunotherapy response

**Figure 7:**
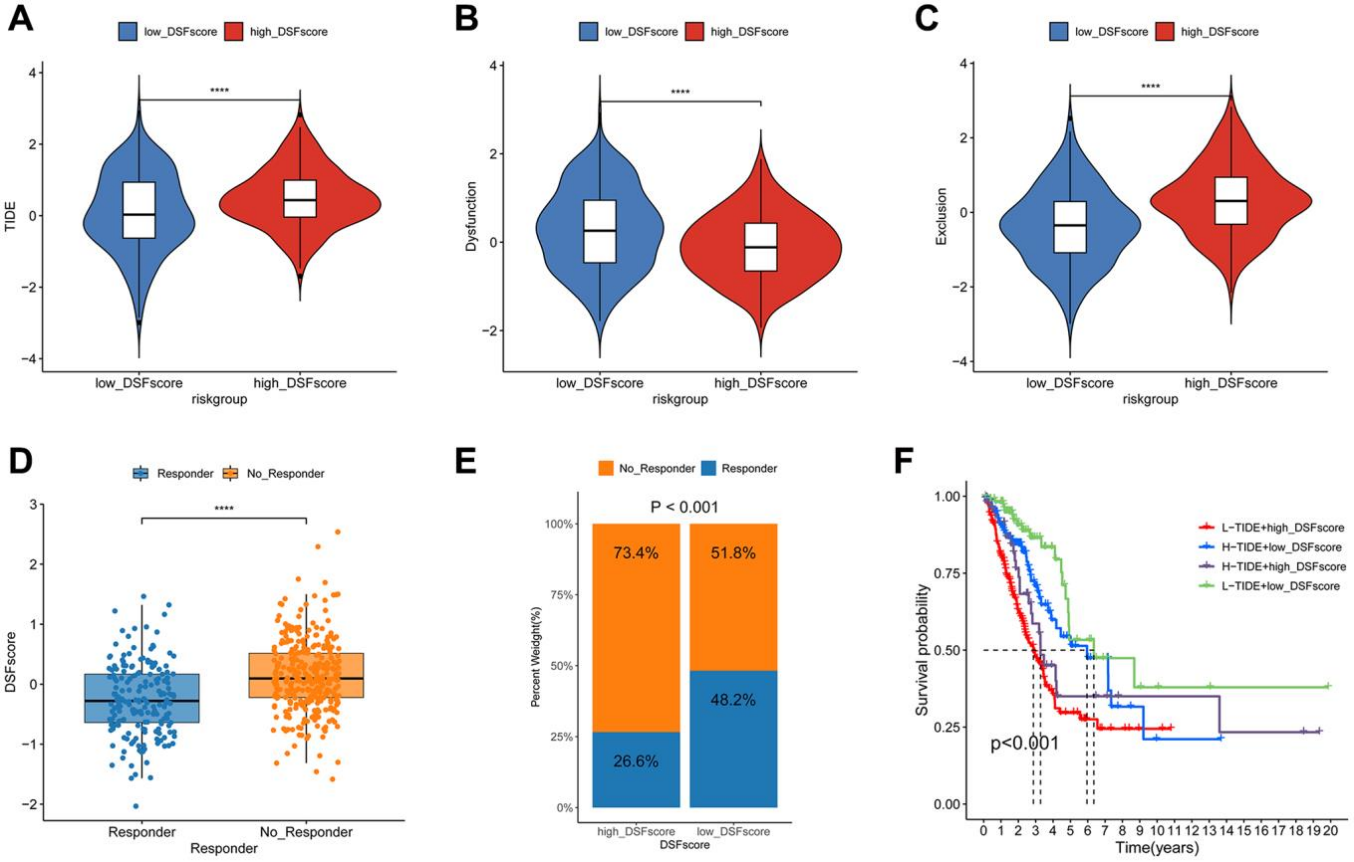
**Estimation of the DRG prognostic model in immunotherapy response.** Difference in TIDE score (**A**), dysfunction score (**B**), and exclusion score (**C**) in two risk groups. (**D**) Assessment of DRG score divergence between responder and non-responder cohorts using the TIDE algorithm. (**E**) The proportion of patients with different response to immunotherapy in two risk groups. (**F**) The Kaplan-Meier OS curves among four groups classified by the DRG score and TIDE score. ^****^P < 0.0001.

We used the TIDE algorithm to predict immunotherapy response in LUAD patients using transcriptomic data. Our results demonstrated that the TIDE score was significantly lower in the low-risk group of patients compared to the high-risk group, indicating a potential increased responsiveness to immunotherapy in the low-risk group (Figure 7A). Furthermore, the low-risk cohort displayed an increased immune dysfunction score along with a reduced immune exclusion score, as shown in Figure 7B, 7C, respectively. Employing the TIDE algorithm, we distinguished immunotherapy responders from non-responders and noted that responders were linked to a lower DRG score (Figure 7D, 7E). Patients with both a high DRG score and a low TIDE score demonstrated the worst prognosis, as depicted in Figure 7F. Our study highlights the clinical potential of the TIDE algorithm in guiding immunotherapy decisions for LUAD patients. Furthermore, we included a GEO cohort for immunotherapy validation, with results presented in Supplementary Figure 2. Our risk signature also demonstrates strong validation performance.

### Correlations of DRG score with CRSGs and chemotherapeutic sensitivity

**Figure 8:**
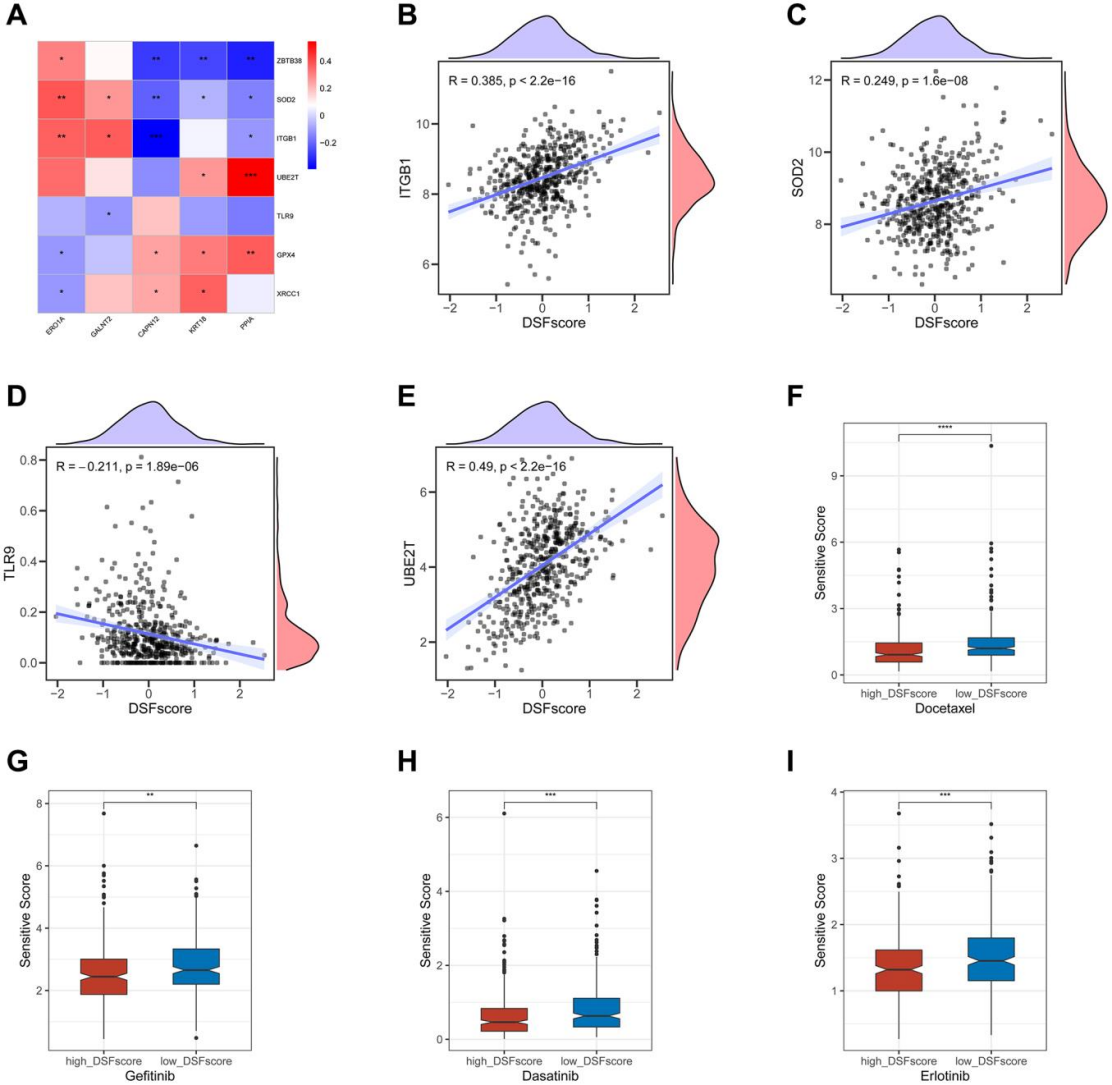
**Correlations of the DRG score with CRSGs and chemotherapeutic sensitivity in LUAD.** (**A**) Correlations between the expression of CRSGs and five genes in the DRG prognostic model. (**B**–**E**) Correlations between the expression of CRSGs and the DRG score. (**F**–**I**) Difference in chemotherapeutic sensitivity between high- and low-risk groups. ^*^P < 0.05, ^**^P < 0.01, ^***^P < 0.001, ^****^P < 0.0001.

Our findings have shown a significant association between the expression levels of CRSGs and those of ERO1A, KRT18, GALNT2, PPIA, and CAPN12 (Figure 8A). Furthermore, we observed that as the DRG score increased, there was a corresponding increase in the expression levels of ITGB1, SOD2, and UBE2T, while a simultaneous decrease was observed in the expression levels of TLR9. This phenomenon suggests a potential connection between the DRG score and the effectiveness of chemoradiotherapy (Figure 8B–8E). To evaluate whether the DRG score could serve as a promising biomarker for predicting chemotherapy response in LUAD patients, we performed a thorough drug sensitivity analysis using the “oncoPredict” R package. Our results indicated that patients in the high-risk group had lower estimated sensitivity scores for docetaxel, gefitinib, dasatinib, and erlotinib (Figure 8F–8I), suggesting that patients with a high DRG score were more likely to benefit from chemotherapy.

### Single-cell sequencing characterization of the TME in LUAD

**Figure 9:**
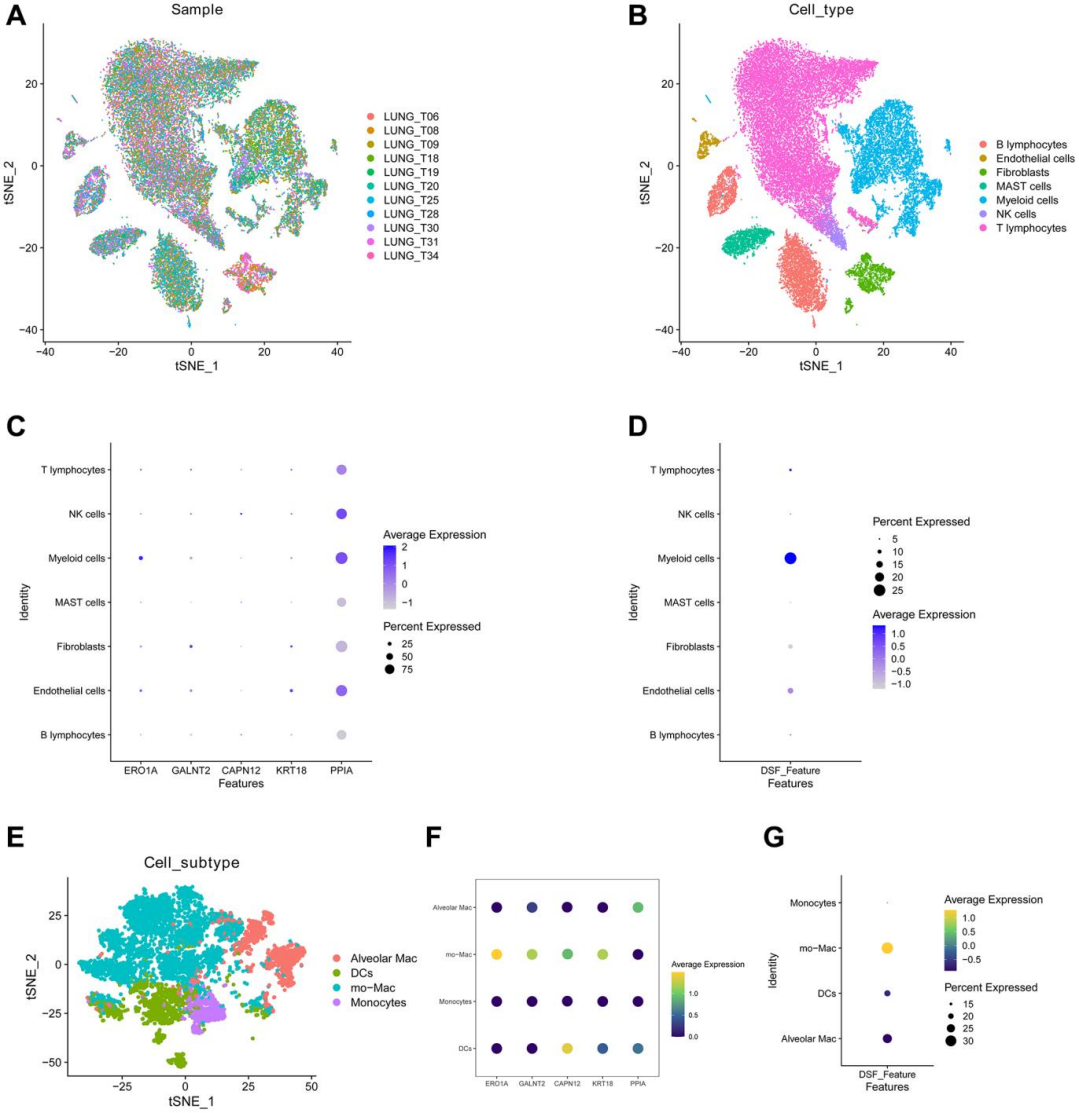
**The distribution of the DRG score in tumor microenvironment.** (**A**) The integration performance of the 11 samples exhibits a high degree of efficacy. (**B**) tSNE plot showed seven cell types from 37,364 cells. (**C**) The different expression of hub genes in seven cell clusters. (**D**) The DRG score exhibited a predominant distribution within the myeloid cellular population. (**E**) Myeloid cells were classified into alveolar macrophages (Alveolar Mac), dendritic cells (DCs), monocyte macrophages (mo-Mac), and monocytes. (**F**) Distinctive characteristics of hub genes across four myeloid cell types. (**G**) DRG score was primarily manifested in mo-MAC.

Recently, single-cell RNA sequencing has emerged as a powerful technique for characterizing the molecular characteristics of individual cells. This technology enables a precise understanding of the TME by facilitating the analysis of the transcriptomic landscape of specific cells [10]. To explore the role of the DRG score in the TME, we conducted a more comprehensive study by extracting and refining TME data obtained through single-cell RNA sequencing. Our analysis of 11 samples revealed successful integration without any noticeable batch effects, rendering them suitable for further analyses (Figure 9A). Utilizing the tSNE algorithm, we identified and labeled seven distinct cell clusters among the 37,364 quality-controlled cells based on their lineage marker genes. The primary annotated cellular clusters included various types of immune cells, such as B lymphocytes, myeloid cells, natural killer cells, and T lymphocytes (Figure 9B). We examined the expression patterns of key genes in seven distinct cell clusters. Our results show that PPIA had significantly increased expression in NK cells, myeloid cells, and endothelial cells, whereas ERO1A predominantly showed high expression levels in myeloid cells (Figure 9C). Additionally, the DRG score was predominantly enriched in myeloid cells (Figure 9D). To investigate the potential association of hub genes with myeloid cells, we categorized them into four distinct subgroups: alveolar macrophages (Alveolar Mac), dendritic cells (DCs), monocyte macrophages (mo-Mac), and monocytes (Figure 9E). Our analysis results have shown that the Alveolar Mac clusters have demonstrated increased expression levels of PPIA, whereas the DCs clusters have shown elevated expression levels of CAPN12. Furthermore, the majority of hub genes (4 out of 5) showed statistically significant upregulation in mo-MAC cells, except for PPIA (Figure 9F). Importantly, the DRG score was mainly observed in mo-MAC cells, as opposed to DCs and alveolar Mac cells (Figure 9G).

### Validation of the prognostic model

**Figure 10:**
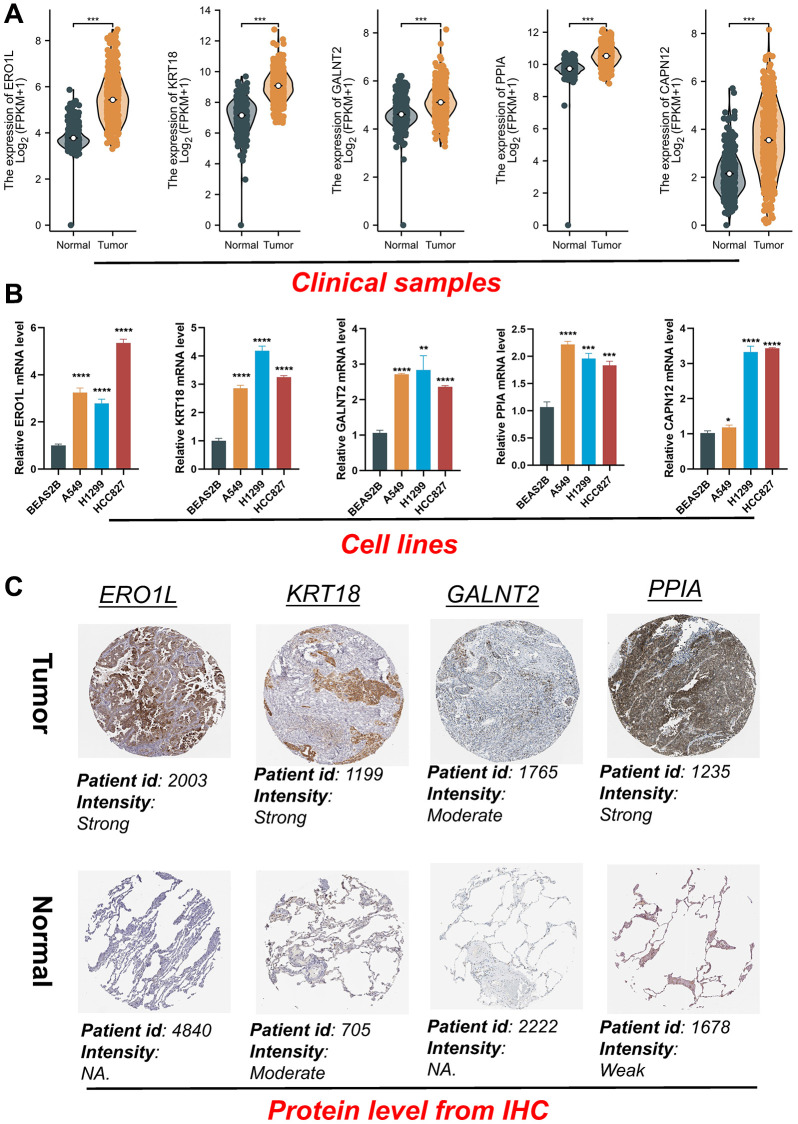
**Validation of the expression patterns of five signature genes.** (**A**) The gene mRNA expressions of five signature genes in the normal and tumor tissues. (**B**) Five signature genes expression in normal and LUAD cell lines. (**C**) Immunohistochemistry of the ERO1L, KRT18, GALNT2, PPIA in the normal and tumor groups from the HPA database. ^*^P < 0.05, ^**^P < 0.01, ^***^P < 0.001, ^****^P < 0.0001.

To further demonstrate the feasibility of the prognostic model, we conducted qRT-PCR testing on LUAD cells, RNA-seq analysis on clinical tissues, and protein content testing at the IHC level. Initially, we integrated the GTEx database, which contains normal tissues, and the TCGA database, which contains tumor tissues, to examine mRNA expression levels. Our investigation showed a significant increase in the expression levels of the five signature genes in tumor tissues compared to normal tissues (Figure 10A). Furthermore, we validated the expression profiles of the five genes relevant to the proposed model. The investigation employed three distinct LUAD cell lines: A549, H1299, and HCC827, in addition to a normal bronchial epithelial cell line, BEAS2B. The analysis findings were consistent with data obtained from RNA-seq of clinical tissues (Figure 10B). Furthermore, in the IHC slices from the HPA database, these genes exhibited higher protein expression in most tumor samples compared to normal bronchial epithelial tissues, showing stronger staining. However, CAPN12 did not have corresponding antibodies (Figure 10C). Overall, the presented findings reinforce the stability and reliability of the risk signature.

## DISCUSSION

Cell death is crucial for maintaining an organism’s balance, fostering its growth and development, and preventing hyperproliferative and malignant disorders from arising [11, 12]. Recently, researchers identified a previously unknown type of cell death called disulfidptosis, which differs from the known forms of programmed cell death (ferroptosis, pyroptosis, necroptosis, and cuproptosis). Cellular death in this manner is triggered by disulfide stress resulting from an excess of cysteine, and it has been observed to occur in situations marked by a lack of glucose availability [7]. Up to now, no studies have specifically investigated the connection between disulfidptosis and the TME, or its impact on immunotherapy.

Using the gene expression profiles of DRGs, we successfully identified two distinct molecular clusters associated with disulfidptosis in the TCGA-LUAD cohort. Patients diagnosed with LUAD in the C2 subgroup displayed reduced survival rates and presented advanced clinicopathological characteristics. It is reasonable to speculate that these differences may partly arise from different responses to therapeutic treatments. In line with the aforementioned assumption, we confirmed significant variations in the expression of therapy-associated genes among distinct molecular clusters, which include CRSGs and ICGs. This suggests that the therapeutic response differs among DRG clusters, with C1 showing an up-regulated expression profile in ICGs and C2 in CRSGs. Furthermore, C1 exhibited higher values in immune, stromal, and ESTIMATE scores compared to C2. Previous studies in the context of LUAD have shown that neoplastic lesions with distinctive TIME profiles are likely to demonstrate different responses to both chemotherapy and immunotherapeutic treatments [13–15].

An increasing amount of evidence has demonstrated the role of disulfides in initiating and advancing tumors at the transcriptomic level [16]. During our investigation, we conducted a thorough examination of mRNA transcriptome variations within distinct disulfide patterns. We identified 1,421 DEGs associated with the DRG cluster. To enhance the assessment of the disulfidptosis pattern in LUAD patients, we created a DRG prognostic model incorporating ERO1A, KRT18, GALNT2, PPIA, and CAPN12. Endoplasmic reticulum oxidoreductin 1-α (ERO1A) plays a crucial role as a regulator of protein disulfide isomerase, and recent research has suggested that both protein disulfide isomerase and ERO1A are significant factors in tumor prognosis [17]. KRT18, also referred to as CK18, is a cytoskeletal protein. Dysregulation of KRT18 is linked to the development and progression of a wide range of cancers [18]. Knocking down KRT18 in NSCLC reduces cell migration and significantly enhances chemotherapy sensitivity [19]. N-Acetylgalactosaminyltransferases (GALNTs) form a family of glycosyltransferases responsible for synthesizing mucin-type O-glycans. GALNT2, among these family members, is notably overexpressed in NSCLC tissues, and its overexpression is strongly correlated with a poor prognosis. Downregulating GALNT2 has been shown to hinder the proliferation, migratory potential, and invasiveness of NSCLC cells. Simultaneously, it exerts a pro-apoptotic effect and triggers cell cycle arrest [20]. In a recent study conducted by Jia et al., it was found that PPIA, an immune-related gene, can serve as a potential prognostic biomarker for predicting lymph node metastasis and prognosis in lung adenocarcinoma [21]. Emerging evidence suggests that signature genes may play important roles in lung cancer. In this investigation, we formulated a risk assessment system that categorizes LUAD patients into either a high-risk or low-risk group using signature genes. Our analysis revealed a significant difference in clinical outcomes between the high-risk group and the rest of the patients, as demonstrated in both the TCGA training and testing datasets. Furthermore, ROC curves confirmed the outstanding predictive performance of the signature in evaluating the prognosis for individuals with LUAD over 1-year, 3-year, and 5-year periods. The distribution plots and Kaplan-Meier curves confirmed a decrease in overall survival time with increasing DRG scores in both the TCGA training and testing cohorts. Furthermore, our study showed that the nomogram provided improved clinical utility for predicting the outcome of patients with LUAD compared to individual prognostic factors. Overall, the findings indicate that the DRG score is an independent prognostic factor for predicting the outcome of LUAD patients.

Despite the significant progress made in the therapeutic management of LUAD over the past decade, there is still a compelling need for further advancements in this field. Various standard-of-care multimodal treatments, such as surgery, radiation therapy, immunotherapy, and chemotherapy, have been proposed. However, the effectiveness of these treatments is constrained by the diversity in treatment responses and outcomes among LUAD patients [22]. Specifically, the pathological characteristics of LUAD, such as mutations and resistance to therapy, play a crucial role in influencing the clinical response to treatment [23]. Therefore, further research is crucial for obtaining a better understanding of the prognostic implications and treatment responses related to intratumoral heterogeneity and TMB in LUAD patients. In line with previous research, our findings show a notable difference in TMB levels among various subgroups categorized by DRG scores. Patients with elevated DRG scores had a greater chance of carrying essential gene mutations, especially in the TP53 gene. Earlier studies have already confirmed a positive connection between high TMB scores and enhanced response to immunotherapy. In our present analysis, we observed a significantly higher TMB score in the high-risk group and discovered a positive correlation between DRG score and TMB score. Given the possible connection between TMB and a positive clinical response to immunotherapy, we carried out a comprehensive investigation to evaluate the effectiveness of immunotherapy in different LUAD subpopulations. Our study found 52 ICGs that showed differential expression in the five signature genes. Furthermore, our investigation uncovered a positive correlation between the DRG score and the biomarkers CD276, PVR, and KIR2DL4. This discovery emphasizes the potential usefulness of these biomarkers as valuable predictors of treatment responses in a clinical setting. In this study, we employed the TIDE algorithm to predict the effectiveness of immunotherapy in LUAD patients, using transcriptomic data. Our results showed a significant increase in the TIDE score in the high-risk group compared to the low-risk group. These results strongly suggest that patients in the low-risk group may obtain more significant clinical benefits from immunotherapy interventions. Importantly, patients in the low-risk group demonstrated higher dysfunction scores and lower exclusion scores. Furthermore, utilizing the TIDE algorithm, we identified individuals responding and not responding to immunotherapy and observed that responders were linked to lower DRG scores. Taken together, these observations suggest that individuals classified as low-risk may have increased susceptibility to immunotherapeutic interventions. Furthermore, we examined the relationship between disulfidptosis patterns and the response of LUAD patients to radiation and chemotherapy. Our findings have shown a positive correlation between the expression levels of CRSGs and the DRG score. Targeting DRGs can enhance therapeutic outcomes. In particular, our study demonstrated that patients with a high DRG score exhibited increased responsiveness to docetaxel, gefitinib, dasatinib, and erlotinib, which suggested improved clinical results. Based on our research findings, it is clear that the DRG score holds the potential to serve as a reliable prognostic marker for forecasting the effectiveness of immunotherapy and chemotherapy in LUAD patients.

Single-cell RNA sequencing has emerged as a powerful technology for characterizing the molecular features of individual cells, enabling a highly accurate understanding of the TME [10, 24]. To investigate the role of the DRG score in the TME, we conducted a more detailed study by extracting and refining TME data from single-cell RNA sequencing. Our findings indicate that PPIA was significantly upregulated in NK cells, myeloid cells, and endothelial cells, while ERO1A exhibited high expression levels primarily in myeloid cells. Moreover, the DRG score exhibited significant enrichment in myeloid cells. In order to explore the possible relationship between hub genes and myeloid cells, we categorized them into four distinct subgroups: alveolar macrophages (Alveolar Mac), dendritic cells (DCs), monocyte macrophages (mo-Mac), and monocytes. Our analysis revealed a significant increase in PPIA expression within Alveolar Mac clusters, along with a notable elevation in CAPN12 expression within DCs clusters. Furthermore, the majority of the hub genes (4/5) exhibited significant upregulation in mo-MAC cells, with the exception of PPIA. Importantly, the DRG score was primarily observed in mo-MAC cells, as opposed to DCs and alveolar Mac cells.

However, despite the positive findings, several unresolved issues demand our attention. First and foremost, we must recognize that the retrospective creation of the DRG risk signature was based on publicly available databases, potentially introducing inherent selection biases. To determine the generalizability and robustness of our results, it is crucial to conduct extensive prospective and multicenter clinical investigations. Moreover, it is essential to recognize that several key clinical factors, such as chemoradiotherapy and surgery, were not included in the datasets being analyzed. As a result, their incorporation in future studies is imperative. This limitation might have impacted the accuracy of the analyses related to treatment response and disulfidptosis state. Additionally, to validate the expression of signature genes, it is essential to include a larger number of clinical pathology samples. Furthermore, conducting additional experiments *in vivo* and *in vitro* is crucial to comprehensively understand the roles of signature genes within the disease’s context.

## CONCLUSIONS

In summary, our study offers a comprehensive analysis of DRG expression profiles in LUAD and introduces a novel risk model for evaluating therapy response and patient prognosis. This model, in particular, relies on a 5-DRG signature of genes (ERO1A, KRT18, GALNT2, PPIA, and CAPN12), and we have explored these genes’ roles in LUAD, considering their impact on the tumor’s immune microenvironment, clinical characteristics, prognosis, and therapy approaches. The findings of this study are clinically significant and suggest that disulfidptosis may be a potential therapeutic target for individuals with LUAD.

## Supplementary Materials

Supplementary Figures

Supplementary Tables
