# Supplementary material for: Integrative analysis of single-cell and bulk RNA-sequencing data revealed disulfidptosis genes-based molecular subtypes and a prognostic signature in lung adenocarcinoma
Source: Aging (Albany NY). 2024 Feb 5;16(3):2753–73. doi: 10.18632/aging.205509 (PMC10911368; doi:10.18632/aging.205509)
Supplement: Supplementary Tables [file aging-16-205509-s002.pdf]

SUPPLEMENTARY TABLES

Supplementary Table 1. Summary of 10 disulfidptosis-related genes.

Gene
GYS1
NDUFS1
OXSM
LRPPRC
NDUFA11
NUBPL
NCKAP1
RPN1
SLC3A2
SLC7A11

Supplementary Table 2. Chemoradiotherapy sensitivity–related genes.

Gene	Doi	Characteristic
ITGB1	https://doi.org/10.7150/ijbs.52319	Negative
XRCC1	PMCID: PMC8290768	Negative
TLR9	https://doi.org/10.1016/j.ccell.2021.12.009	Positive
ZBTB38	https://doi.org/10.1186/s12967-022-03372-0	Negative
UBE2T	https://doi.org/10.1016/j.canlet.2020.06.005	Negative
GPX4	https://doi.org/10.3389/fonc.2022.913669	Negative
SOD2	https://doi.org/10.1007/s00228-015-1824-0	Negative

Supplementary Table 3. Primer sequences for mRNAs.

Species	Gene	Primer Sequence	
Homo sapiens	ERO1L	Forward	GGCTGGGGATTCTTGTTTGG
		Reverse	AGTAACCACTAACCTGGCAGA
Homo sapiens	KRT18	Forward	TGGAAACCCAGCTCTGACTC
		Reverse	TGGGGCTTTCTTGGTCTTCT
Homo sapiens	PPIA	Forward	GGTGGTTCGTGGTGAACG
		Reverse	AGCTTGTTGTCCACAGTCAGCAAA
Homo sapiens	GALNT2	Forward	GCTGGGCATCGCCTACTAC
		Reverse	GGTTAAAGTCTGGCCACCGT
Homo sapiens	CAPN12	Forward	ACTGACCTCCTTCTTGGTGC
		Reverse	GTGGCCAAGGTAGCAGCTTA
